# Non-Targeted Analysis of Carbofuran and Related Compounds in Commercial Formulations and Animal Tissue

**DOI:** 10.3390/molecules31020259

**Published:** 2026-01-12

**Authors:** Genny Grasselli, Adriana Arigò, Giorgio Famiglini, Zdena Skrob, Arthur Sniegon, Tomas Cajthaml, Achille Cappiello

**Affiliations:** 1 Department of Pure and Applied Sciences, University of Urbino Carlo Bo, 61029 Urbino, Italy; g.grasselli1@campus.uniurb.it (G.G.); adriana.arigo@uniurb.it (A.A.); giorgio.famiglini@uniurb.it (G.F.); 2 Institute of Environmental Studies, Faculty of Sciences, Charles University, 12801 Prague, Czech Republic; zdena.skrob@natur.cuni.cz (Z.S.); sniegona@natur.cuni.cz (A.S.); tomas.cajthaml@natur.cuni.cz (T.C.); 3 Department of Chemistry, Vancouver Island University, Nanaimo, BC V9R 5S5, Canada

**Keywords:** LC-MS, high resolution, electron ionization, LEI interface, Q-TOF, non-targeted analysis

## Abstract

Recently, some cases of intentional animal poisoning using Carbofuran (CF) occurred in the Czech Republic, although CF is no longer available in the EU market. The present study describes a novel non-targeted analysis (NTA) workflow developed to possibly characterize 13 CF formulations from various sources to be certainly identified in real samples. Furthermore, a detection and quantification method for CF was developed for analyzing three animal samples, obtained from dead animals. The analyses were conducted using the liquid electron ionization (LEI) interface coupled with a quadrupole time-of-flight (QTOF) mass spectrometer, allowing the simultaneous characterization of the formulation’s volatile and low-volatile fractions. Almost all compounds detected in the different formulations were identified by comparing the experimental spectra with the NIST library at high probability values (95–99.38%). Determination of molecular ions, followed by MS/MS analysis, was performed to confirm compound identities at a high level of confidence. The quantification method for CF was successfully validated, showing negligible matrix effects (107%). CF was detected in two out of the three real samples. Only 3-keto-carbofuran was detected in one of the real samples; without any other marker, it was not possible to identify the specific formulation used in the three poisoning cases.

## 1. Introduction

Pesticides play a crucial role in agricultural management, improving crop yield, quality, and safety by acting against pests [1]. Despite these benefits, pesticides often exhibit toxicity toward non-target organisms [2]. Among pesticides, the carbamate class is largely used in agriculture, gardening, forestry, and therapeutic pharmaceuticals. Carbamates are replacing other pesticide classes, such as organophosphate pesticides, because of their shorter half-life and lower persistence and bioaccumulation in the environment [3]. Despite their positive effects, carbamate pesticides show high toxicity for non-target organisms. They bind to the active site of the acetylcholinesterase, inhibiting the enzyme, causing an accumulation of acetylcholine at the nerve synapse. The inhibition of acetylcholine hydrolysis leads to increased cholinergic stimulation culminating in poisoning symptoms [4]. In addition, they are suspected carcinogens and mutagens [5].

Carbofuran (CF) is a carbamate systemic insecticide, nematicide, and acaricide largely used for its broad spectrum of activity. Because of its high mobility in different soils and high solubility in water, CF has been detected in high concentrations in rivers, food, and soils worldwide [3]. The European Union banned carbofuran in 2008 due to its toxicity [6], yet it remains available in various countries [7]. Different accidental and intentional animal poisonings have been documented over the years in different countries, such as Spain [8], Romania [7], South Korea [9], and so on. In addition, CF has been used to commit human suicide [10].

Cases of intoxication and fatalities caused by carbamate pesticides were mainly investigated by gas chromatography (GC) and liquid chromatography (LC) coupled with mass spectrometry (MS) [11,12,13,14,15]. In forensics, LC-MS is commonly used for targeted analysis. Soft ionization techniques coupled with LC, particularly electrospray ionization (ESI), offer high sensitivity, allowing the ionization of thermolabile, high-molecular-weight, and non-volatile compounds. In addition, derivatization is not required, thus reducing sample preparation time. However, ESI produces mainly molecular ions, providing less structural information compared to electron ionization (EI), hence making it more difficult to determine unknown structures [16]. To enhance specificity, analyses are usually conducted in MS/MS mode [16]. Despite their widespread use, no universal spectral library has been created for unknown compound identifications in LC-MS due to the variability of ionization conditions, which are influenced by different instrument types, polarity modes, and mobile phases composition [17,18]. High-resolution mass spectrometry (HRMS), combined with different chemical databases, is now the best technique for unknown compounds identification. Instruments such as Orbitrap and Quadrupole Time-of-Flight (QTOF) enable HRMS in both LC and GC applications [4,10,19,20,21]. Both instruments achieve the required mass accuracy (below 5 ppm) and absolute isotope ratio deviation (within 5%) for accurate structure elucidation. However, factors like ion count levels, complex sample matrices, and compound coelution in LC can sometimes compromise accuracy [22,23,24]. Considering the high identification power, HRMS is the most used technique in non-targeted analysis (NTA) [25]. A generic NTA workflow includes different steps that have to be optimized; these are sampling, analysis, data pre-processing, prioritization, and identification [25]. Sampling and analyses should be planned based on the chemical/physical properties of substances; in this case, the sample preparation and chromatographic separation techniques used can strongly affect the attendance of the results [25]. The data pre-processing step is fundamental in cases of high data complexity, including peak detection, annotation, and background subtraction. It includes several procedures, such as establishing an S/N threshold of 10 [26], and deconvolution [27]; different commercialized softwares are currently available to make these procedures easier [24,28]. In the case of a complex study, such as when samples have to be grouped along a time or space gradient, the alignment of components is performed by statistical methods, allowing the prioritization of the most relevant components. Depending on the type of study, algorithms can help in this process [29]. In the end, prioritized compounds are identified using all the tools dispensed by the instruments and software. In order to evaluate the attendance of the identification, different levels of confidence were proposed by Schymanski et al. [30]. Levels range from 1 to 5, where level 1 is reached when the proposed structure is also confirmed with RT matching injecting the standard, while level 5 corresponds to the assignment of only extract mass. Since level 1 is hardly reached due to the standard not always being available, level 2a can be easily reached using EI due to the possibility of comparing spectra with libraries. Different studies were published using these levels of confidence as guidance. A work presented by Wurzler and co-workers, aimed at the investigation of poisoning by pesticides in criminal cases, reported the identification of three unknown compounds using ESI-HRMS and DART-HRMS with a level of confidence of 2, obtaining unambiguous matches among MS and MS/MS HR spectra and MS/MS HR library, low mass error, and the detection of diagnostic fragment ions [20]. Another study based on LC-ESI-HRMS for identifying unknown contaminants in recycled textiles also used this confidence level system. In this case, several compounds were identified with confidence levels between 3 and 2b; among them, six substances were matched with persistent, mobile, and toxic (PTM) chemicals established by the European Union (EU) [31]. Liu et al. identified 59 substances in 50 teether toys using GC–GC-Orbitrap MS in EI full-scan mode, with levels of confidence between 2 and 1 [32]. In this case, the analyses were conducted in chemical ionization (CI) as well, proving to be useful for structure determination in case the compounds were not present in the EI library. Another study based on GC-HRMS, presented by Zhang and co-workers, identified 5344 organic compounds with confidence levels between 1 and 2 [33]. Despite the fact that recently the number of LC-MS libraries are rapidly increasing [34], as already mentioned, the use of EI is beneficial due to the possibility of comparing the highly reproducible experimental spectra with library spectra, allowing for the identification of structures with higher levels of confidence.

In 2017, a new interface, called Liquid Electron Ionization (LEI), was developed to combine the advantages of EI with those of LC [35]. LEI interface extends the EI searchable library spectra to compounds not amenable to GC [36,37,38]. Compared to typical LC ionization techniques like ESI [39], the LEI interface demonstrates reduced matrix effects [16,29,40]. Recent studies have shown that LEI is compatible with QTOF, making it a versatile tool [36]. While EI spectra commonly lack measurable molecular ions [25], the QTOF’s low-energy EI capabilities allow the acquisition of high-informative spectra under standard EI conditions and softer fragmentation at lower energies (15 and 9 eV), which yields molecular ions for most compounds. Switching between high and low energies is straightforward, enabling qualitative analyses of unknowns in complex matrices using EI-HRMS.

Recently, some cases of animal poisoning occurred in the Czech Republic, where poisoned food was used to kill multiple animals. The National Veterinary Institute of Prague performed autopsies, finding morphological signs consistent with carbamate pesticide poisoning. The Faculty of Sciences at Charles University in Prague suspected the use of certain CF formulations. Given that CF is banned in the Czech Republic, CF formulations from various countries were provided for analysis using LC-LEI-QTOF-MS/MS. This approach aimed to characterize volatile and low-volatile fractions to identify the specific CF formulation used in the poisonings.

A novel targeted and non-targeted analysis (NTA) method was developed to characterize formulations and detect and quantify CF in three poisoned animal samples. By combining EI with HRMS in LC-LEI-QTOF-MS/MS, several compounds were identified in the formulations, establishing a reliable identification method; this represents the novelty of the work, which allowed the compound identification with high confidence levels due to the triple confirmation of their identities using the same instrument and ion source. The identities were confirmed by NIST comparison, determination of their molecular ions and accuracies, and correlating the structures by comparing the HR-MS/MS spectra with PubChem and ChemSpider. The characterized formulations were compared to real biological samples to trace the source of the CF used in the poisonings, considering the detection even of CF metabolites and unexpected compounds present in the real samples. Due to EI-reduced susceptibility to matrix effects (*ME*), deconvolution allowed the identification of coeluted compounds. In the analysis of the three biological samples, CF poisoning was confirmed in two cases. The method demonstrated satisfactory repeatability, linearity, and negligible *ME* without using an internal standard.

## 2. Results and Discussion

The LC-LEI-QTOF-MS/MS system represents a powerful tool for unknown structure elucidation and identification, as already demonstrated in a previous work [38]. Its ability to generate EI spectra comparable to the NIST library enables rapid and straightforward compound identification. However, when unknown concentrations are too low or coeluting compounds are present, the NIST match can become less reliable. The fast scan speed of the QTOF, combined with the possibility of acquiring HR-MS and HR-MS/MS spectra, enhances the deconvolution of coeluted compound spectra. This process improves background subtraction, permitting a more accurate identification using the NIST library [41].

### 2.1. Characterization of Carbofuran Formulations

In 7 of the 13 formulations (Table 1), different compounds, in addition to CF, were putatively identified using Agilent MassHunter unknown analysis.

**Figure 1:**
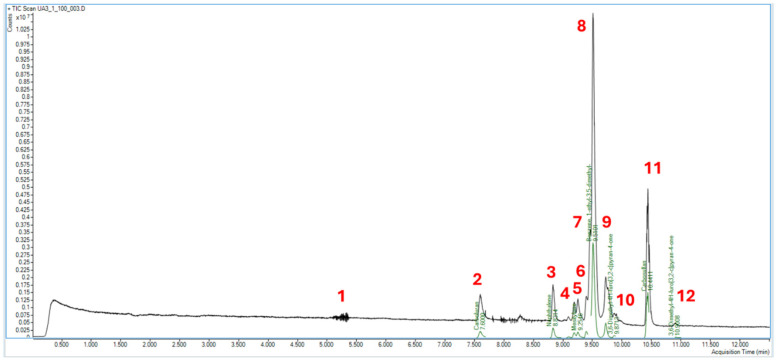
Deconvoluted chromatogram of UA_3 formulation.

*UA_3 Formulation*. In the UA_3 formulation, 12 peaks were detected after deconvolution, as shown in Figure 1 and summarized in Table 2. Eight were readily annotated by matching their spectra with the NIST library (peaks 1, 2, 3, 4, 6, 7, 8, 11). Two compounds were identified with a probability of 57.21% (peak 1) and 76.74% (peak 11). Two compounds gave incorrect matches (peaks 5 and 9), while no matches were proposed for the remaining two (peaks 10 and 12).

**Figure 2:**
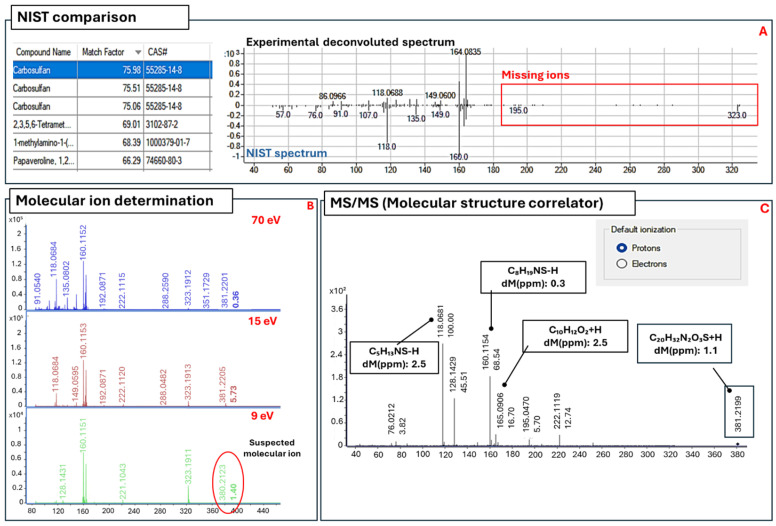
(**A**–**C**). (**A**) Comparison between the deconvoluted spectrum and NIST spectrum of Carbosulfan (peak 11). (**B**) Molecular ion determination comparing the spectra obtained at 70, 15 and 9 eV. (**C**) MS/MS analysis of suspected (M+H)^+^ (*m*/*z* 381.2166) and determination of fragment structures with their corresponding accuracy.

For the compounds identified by NIST library, CF (Figure S1A–C), Naphthalene (Figure S2A–C), Indane (Figure S3A–C), Mesitylene (Figure S4A–C), Indane-1-Methyl (Figure S5A–C), and Cymene (Figure S6A–C), were identified with probabilities ranging between 90.11 and 99.38% and confidence levels between 1 and 2a (Table 2). Dibutylamine and Carbosulfan were identified with a probability below 80%, hence considered not recognized by NIST. To confirm the identity of Carbosulfan, and to assign structures to the unknown compounds 5, 10, and 12, low-energy analyses were conducted at 15 and 9 eV to establish suspected molecular ions. For Carbosulfan, as shown in Figure 2B, the ion at *m*/*z* 381.2266 showed an increment in relative abundance from 0.19% at 70 eV to 5.73% at 15 eV, indicating it as suspected molecular ion. The same ion was selected as the precursor ion for MS/MS analysis, and its high-resolution fragmentation pattern was compared with the ChemSpider database. Using protonation as the default ionization, the compound was confirmed as Carbosulfan, confirming the previous identification by NIST.

**Figure 3:**
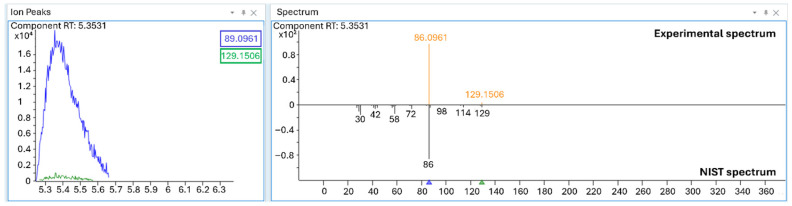
Comparison between experimental deconvoluted spectrum and NIST library spectrum of Dibutylamine (Peak 1).

The ion at *m*/*z* 381.2166 corresponds to the (M+H)^+^ ion of Carbosulfan, as the compound readily gains a proton from the mobile phase, forming the protonated ion under chemical ionization (CI) conditions. In fact, for certain compounds, CI can occur even in an EI source, showing the appearance of the (M+H)^+^ ion [35,38,40]. Comparing the experimental mass with the accurate mass of C_20_H_32_N_2_S+H^+^, the accuracy results in −1.15 ppm, confirming this assumption (Figure 2C). The formation of the (M+H)^+^ is not a reproducible process, hence the relative/absolute abundance of (M+H)^+^ (*m*/*z* 381.2166) and M^+^ (380.2133) results are unstable, producing a decrease in the relative abundance of (M+H)^+^ at 9 eV, differently from what we expected, as shown in Figure 2B. This can be explained because at 9 eV the ionization efficiency is lower, reducing the ionization of the mobile phase and resulting in a reduction in (M+H)^+^ formation compared to M^+^. The presence of Carbosulfan is particularly significant because it was banned by the EU as well [33]. The injection of a less diluted UA_3 formulation (1:10 *v*/*v* instead of 1:100 *v*/*v*) also enabled the detection of Dibutylamine (*m*/*z* 129.1507), peak 1, at RT 5.35, a well-known Carbosulfan degradation product and highly toxic compound [42,43]. The low probability of the match proposed by the NIST library can be ascribed to the compound’s low concentration and because most of the ions fall outside the acquisition range; however, the exact mass of the molecular ion confirms its identity (Figure 3).

Because of the unknown provenience of those formulations, it can be assumed that the active ingredient of the UA_3 formulation was carbosulfan instead of CF. Hence, the presence of CF can be considered a carbosulfan impurity, given that it is considered a photolytic and hydrolysis product of carbosulfan [42,43]. The same procedure adopted for Carbosulfan was applied to peaks 5 and 9, which were not identified by NIST at all. Using low-energy analysis, the suspected molecular ions were determined. The Agilent MassHunter Molecular Structure Correlator software (Version 8.2) (MSC) proposed 2-Methylnaphthalene for peak 5 (Figure S7A,B), and 2,3,6-Trimethylnaphtalene for peak 9 (Figure S8A,B), with a confidence level of 2b.

The same procedure was applied for the identification of peak 10. Also in this case, the comparison with NIST gave an unreliable match. Low-energy analysis showed the ion at *m*/*z* 504.1400 as a suspected molecular ion (Figure S9A,B). In this case, the proposed formula was C_24_H_28_N_2_O_6_S_2_ with an accuracy of 3.3 ppm. Unfortunately, MS/MS analysis could not be performed due to the ion’s low abundance; in this case, the confidence level assigned was 4. The structure of the base peak *m*/*z* 192.0867, present in the C_24_H_28_N_2_O_6_S_2_ spectrum, was also established as C_8_H_18_NS_2_ with good accuracy (−4.26 ppm), confirming once again the proposed formula. Due to its low concentration, it was impossible to identify the molecular ion for peak 12.

*BV_PF_B Formulation.* The second formulation containing another compound besides carbofuran was BV_PF_B (Table 3). In this case, the additional compound was identified as *N*,*N*-diethyl-m-toluamide (DEET), a common mosquito repellent, with a NIST library match probability of 91.84% (Figure S10A). The compound was also confirmed according to the RT by injecting a pure DEET standard, allowing us to reach a confidence level of 1 (Figure S10B).

**Figure 4:**
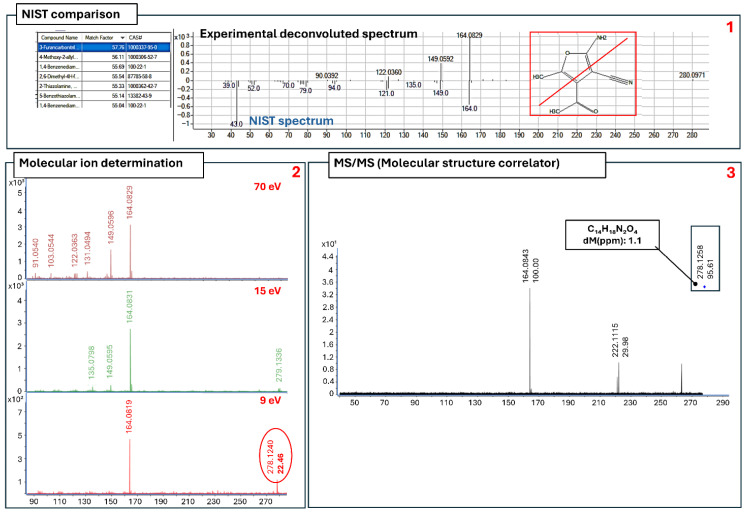
(**1**–**3**). (**1**) Peak 2 molecular formula determination of the CG_23_B formulation. (**2**) Molecular ion determination comparing the spectra obtained at 70, 15, and 9 eV. (**3**) MS/MS of the suspected molecular ion, and its structure and accuracy.

*CG_23_B Formulation*. The third formulation containing a compound other than carbofuran was CG_23_B (Table 4). In this case, the comparison with the NIST library gave a low recognition (probability 57%). In addition, the suspected molecular ion (*m*/*z* 278.1258) (Figure 4), established by performing the low-energy analysis, differed from the compound suggested by the NIST library. Therefore, MS/MS analysis using the *m*/*z* 278.1258 ion as a precursor ion was performed. The MSC software identified the formula C_14_H_18_N_2_O_4_, with an accuracy of −1.1 ppm (confidence level 4).

*F0 Formulation*. In the F0 formulation, a well-known carbofuran metabolite, 3-keto-carbofuran, was detected (Table 5). The detected signal showed very low intensity, which explains the low match value proposed by NIST (Figure S11A–C). Due to the low concentration, it was impossible to determine the molecular ion or perform MS/MS analysis. The metabolite results from the neutral loss of carbamic acid and water [44,45]. In the nine remaining formulations, only carbofuran was found.

### 2.2. Method Validation Parameters

The method for CF was validated by assessing limits of detection (LOD) and quantification (LOQ), intraday and interday repeatability, and linearity range in both solvent and CF-free gastric content of an eagle, used as a matrix. *ME* was evaluated by comparing the slope of the two calibration curves.

**Figure 5:**
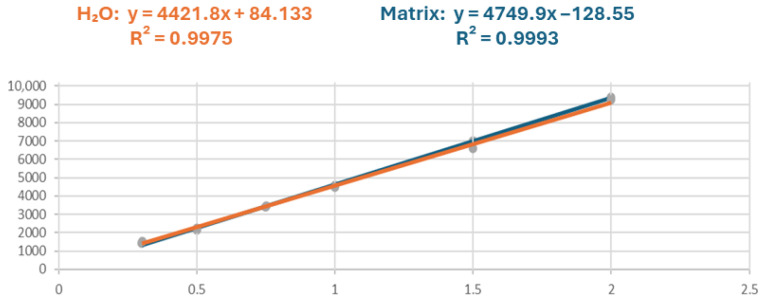
CF Calibration curve in water (orange) and in CF-free gastric content of an eagle, used as matrix (blue).

The LOD and LOQ for CF were 0.09 and 0.30 µg/mL, respectively. Regarding the linearity, R^2^ values of calibration curves in solvent and matrix were 0.9975 and 0.9993, respectively (Figure 5). The RSD% intraday and interday calculated at 0.75 µg/mL were 3% and 10%, and at 1.50 µg/mL were 1% and 9%, respectively.

While previous studies have shown that the LEI interface exhibits negligible *ME*, even with compound coelutions [37,46,47], this work involved analyzing complex real-world samples, necessitating *ME* evaluation. *ME* value was 107%, hence it can be considered negligible, demonstrating the reliability of the method [16].

### 2.3. Real Samples Analyses

**Figure 6:**
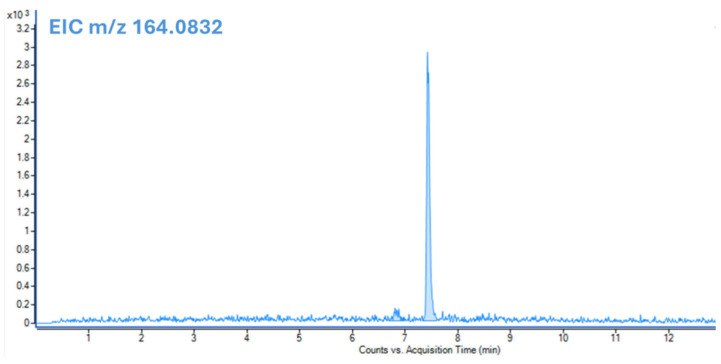
EIC of *m*/*z* 164.0832 Sample 544/2 chromatogram.

**Figure 7:**
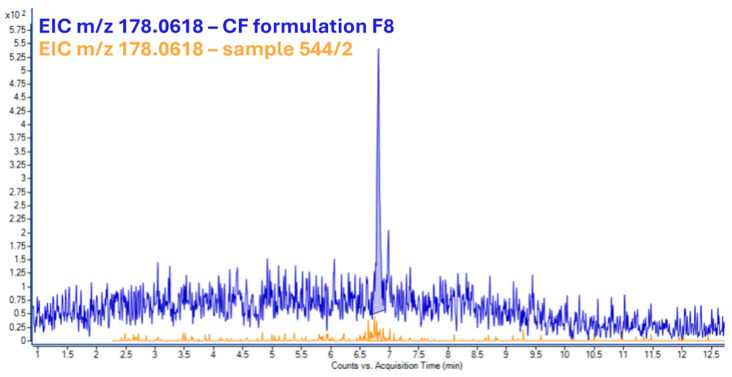
EIC of *m*/*z* 178.0618 (3-Keto-Carbofuran) of F8 formulation (blue) and sample 544/2 (orange).

LC-LEI-QTOF-MS/MS analysis confirmed the presence of CF in two out of the three real samples. In Figure 6, EIC chromatogram of *m*/*z* 164.0832 of the CF is reported. The analyses of the three samples were performed by diluting them with water 1:5 (*v*/*v*). The concentrations of CF found in the extract were 5.5 mg/kg in sample 544/2 (fox liver), 4.2 mg/kg in sample 18894 (red kite gastric content), and below the limit of detection in sample 237 (eagle gastric content). To determine if one of the 13 characterized formulations was used in the poisoning cases, the peak spectra of real samples were compared with our HR laboratory-created library by using Agilent MassHunter Unknown software (Version 10.0). The results showed that 3-keto-carbofuran, a compound found in formulation F0, was also detected in the real sample (554/2), in addition to CF. However, the compound is a degradation product of CF; hence, it cannot be used as a discriminatory marker to trace the specific formulation used (Figure 7).

## 3. Materials and Methods

### 3.1. Chemicals

LC-MS grade acetonitrile (ACN) and methanol were purchased from VWR International, part of Avantor (Milan, Italy). Ultrapure water was obtained from a Direct-Q3 UV water purification system from Merck Millipore Co. (Milan, Italy). Formic acid (FA) (purity 98–100%) was purchased from Honeywell Riedel-de Haën (Seelze, Germany). VWR International (Milan, Italy) provided 1.5 mL vials. PEEK-coated fused silica capillaries were purchased from IDEX (Oak Harbor, WA, USA); fused silica capillaries were from Molex Polymicro (Lisle, IL, USA); flexible stainless-steel tubing was from Agilent Technologies, Inc. (Santa Clara, CA, USA).

CF standard (purity ≥ 98.0%) was obtained from Honeywell Riedel-de Haën. Naphthalene (purity ≥ 99.7%) analytical standards were purchased from Merck (Milan, Italy).

CF formulations and the real samples, one liver from a fox, and the gastric content of an eagle and a red kite, were provided by the Faculty of Science of Charles University, Prague.

### 3.2. Standard Solutions, Formulations, and Real Samples Preparation

CF and naphthalene stock solution were prepared at a concentration of 500 mg/L in MeOH/H_2_O 80:20 (*v*/*v*) and methanol, respectively. The working solutions were prepared daily by diluting the stock solution with ultrapure H_2_O. A total of 13 formulations of CF were provided in granular and liquid forms. The 10 granular formulations were extracted by adding 600 μL of two different solvents, ultrapure H_2_O and MeOH, to about 0.06 g of formulation granules. MeOH did not extract additional compounds compared to ultrapure H_2_O. Therefore, ultrapure H_2_O was preferred, as it also allows for higher injection volumes. Granules were sonicated for 10 min and filtered using 4 mm syringe filters PTFE 0.2 μm (Thermo Fisher Scientific, Waltham, MA, USA). For the three liquid formulations, the samples were diluted 1:100 *v*/*v* using both MeOH and ultrapure H_2_O and filtered using a 4 mm syringe with PTFE 0.2 μm filters. In this case, MeOH was preferred because of the presence of hydrophobic compounds, especially in UA_3 formulation. Regarding the real samples, the biological specimens were freeze-dried, homogenized, and extracted using pressurized liquid extraction (PLE) before the analysis. An already validated PLE procedure, used for the extraction of pesticides from animal foods, was performed using the Dionex ASE system (Thermo Fisher Scientific, Waltham, MA, USA) [48]. The focus of this work was the detection of characteristic compounds to use as markers of each formulation in real samples. Therefore, the extraction procedure was designed to be as non-selective as possible. MeOH was used as the extraction solvent. The samples were mixed with 10 g of sea sand, used as in-cell adsorbent to minimize interference, and put in stainless steel cells. Extractions were performed using MeOH at 80 °C and 1500 psi for three cycles, with 5 min static periods. The program also included 5 min of preheat, 5 min of heating, and a 60 s purge. Methanolic extracts were collected under a nitrogen stream. To remove lipids, a two-step sample clean-up was applied to each extract. The purification was performed using Captiva EMR lipid columns (Agilent Technologies, Santa Clara, CA, USA). Before lipid cartridge loading, 2 mL of each sample were transferred to Eppendorf tubes and centrifuged for 10 min at 14,000 rpm. Supernatants were then transferred to the cartridges; 10 min after loading, 800 µL of MeOH:H_2_O 80:20 solution were added to elute the extracts [49]. For qualitative and quantitative analysis, real samples were injected both undiluted and diluted with H_2_O [49]. The water content in the real samples and standard solution was carefully evaluated as it significantly influenced peak width. Higher water content yielded better chromatographic peak shapes, even with larger injection volumes (up to 20 μL), enabling higher S/N compared to undiluted samples. The best results were obtained by injecting samples with more than 74% of water content.

### 3.3. Method Validation

The calibration curves were constructed by analyzing standard solutions prepared in ultrapure H_2_O and in the sample matrix at six concentrations: 0.3, 0.5, 0.75, 1, 1.5, and 2 mg/L. For the calibration curve in water, a standard solution at a concentration of 5 mg/L was diluted with ultrapure H_2_O. For the calibration curve in the sample matrix, the CF-free gastric content of an eagle was used as matrix, and 20 μL of the matrix was spiked with 80 μL of the standard solutions. Also, for calibration curves in both solvent and sample matrix, the solutions were prepared keeping the water content higher than 74%. Each concentration was injected four times. The two calibration curves were compared for *ME* evaluation; the *ME* was calculated using the following formula:

$$ME(\%)=\frac{\left(Slope\;matrix\right)}{\left(Slope\;solvent\right)}\times 100$$

The limit of detection (LOD) and limit of quantification (LOQ) were determined by injecting five replicates at concentrations corresponding to signal-to-noise ratios (S/N) of 3 and 10, respectively. Intraday repeatability was assessed by performing 10 injections on the same day, while interday repeatability was determined with five injections for five consecutive days. Both repeatability assessments were performed at two concentrations: 0.75 and 1.5 mg/L.

### 3.4. LC-LEI-QTOF-MS/MS Analysis

**Figure 8:**
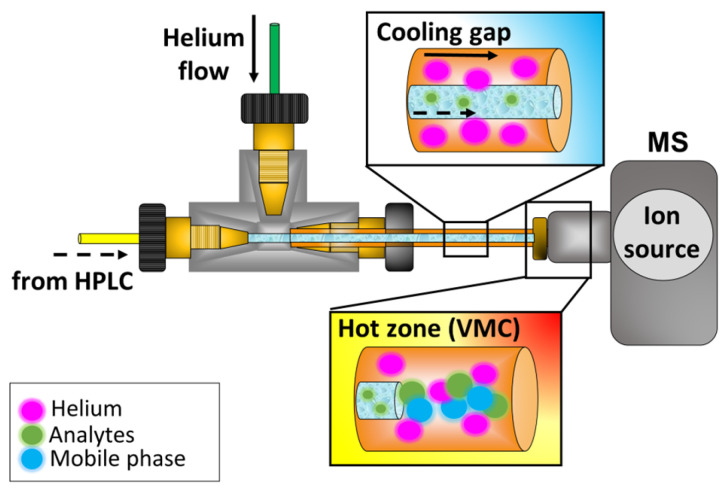
Scheme of LEI interface.

The analyses were conducted using the LEI interface coupled to a conventional QTOF Agilent 7250 MS (Agilent Technologies Inc., Santa Clara, CA, USA), equipped with an EI ion source (Figure 8). More details regarding the interface description are reported elsewhere [40]. Briefly, the Agilent 1290 Infinity II binary pump (Agilent Technologies Inc., Santa Clara, CA, USA) was used for the LC separations, performed in reversed-phase liquid chromatography (RPLC) using a Kinetex 1.7 μm XB-C18 150 × 2.1 mm column. The following elution gradient was applied (mobile phase A: H_2_O + 0.1% FA; mobile phase B: ACN + 0.1% FA): 5% B for 1 min; from 5% B to 100% B in 6 min, 100% B for 7 min. The flow rate was set at 200 µL/min and the injection volume at 20 μL. A Passive Flow Splitter (PFS) reduced the flow rate after chromatographic separation, making it compatible with the LEI interface (500 nL/min). More detailed information on the splitting device is reported elsewhere [49,50]. The split ratio was set at 1:400. Mass spectrometry conditions were as follows: vaporization microchannel (VMC) 350 °C; ion source temperature 260 °C; quadrupole temperature 150 °C; LEI helium flow 1.2 mL/min; quenching flow (helium) 4 mL/min; collision flow (nitrogen) 1 mL/min. TOF calibration was performed before each analysis. Agilent Mass Hunter GC/MS acquisition software (Version 10.1.49.0) was employed to acquire all data. Mass spectra were acquired in full-scan mode using 70 eV, 15 eV, and 9 eV electron energies, and in targeted MS/MS, using the molecular ion as precursor ion. Regarding full-scan analysis, the mass range was set from 85 to 700 *m*/*z* to eliminate low-mass background noise given by the mobile phases. The emission current was set at 4.4 µA for 70 eV, and at 0.6 µA for 15 eV and 9 eV. The acquisition rate was set at 8.00 spectra/s for all analyses.

### 3.5. Software and Database

Agilent MassHunter Qualitative Analysis software (Version 10.0) was employed for data processing using a 5 mg/L extraction window (Figure 2). Using the “Find Compounds by Integration” algorithm, the software identified compounds through MS/MS peak integration, after which the software generated possible formulas for each compound. The resulting .csv files, generated by Agilent Mass Hunter Qualitative Analysis software, containing MS/MS fragmentation data obtained at different collision energies and the corresponding molecular formula information, were exported to Agilent MassHunter Molecular Structure Correlator software (Version 8.2) for formula confirmation and possible structures determination. The software compares the compound formulas with the ChemSpider database, giving a score for each possible structure. The scores are assigned considering each MS/MS fragment ion possible structures; three factors contribute to the definitive score: the “systematic bond-breaking” approach, the mass accuracy of each fragment, and the match of the ion intensities with the candidate structures [49].

Agilent MassHunter Unknown software (Version 10.0) was used to create HR-lab made library.

**Figure 9:**
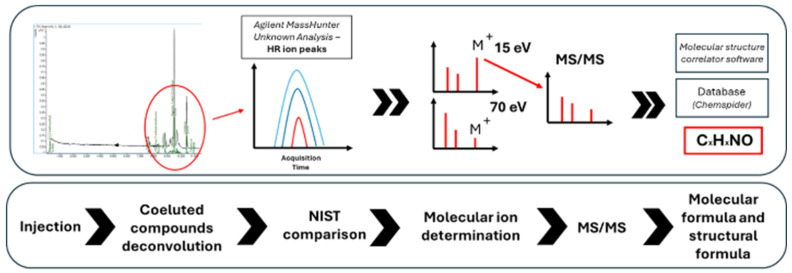
The workflow developed for compound identifications used for the characterization of formulations.

The experimental workflow is reported in Figure 9. 

### 3.6. Non-Targeted Workflow Quality Control

All the analyses were conducted in triplicate and blank analyses were performed after each one by injecting ultrapure water. Regarding the characterization of formulations, only the peaks showing S/N higher than 3 were considered as compounds. S/N was calculated on the extracted ion chromatograms (EIC) of the base peaks. The comparison with the NIST library was performed after the deconvolution using Agilent MassHunter unknown software. The deconvolution ensures obtaining “clean” spectra from background noise as well. Only spectra that show match factors with the library spectra higher than 75% were considered recognized by the library.

For compound structure determination after MS/MS analysis, only results provided by the MSC software showing correlation scores higher than 70 were considered reliable. Regarding the structures proposed with lower scores, only the molecular formula with an accuracy up to 5 ppm was considered reliable. For CF and naphthalene, the compound identification was confirmed by injecting the standards. In this case, both the match between the two spectra and between the retention time of the standard and the compound present in the sample were evaluated.

## 4. Conclusions

The use of EI as ionization technique makes the identification process easier with respect to the conventional ionization techniques used with LC-MS/MS. However, being that EI is usually coupled with GC, the chemical coverage is limited to volatile compounds. Coupling LC with EI allows to extend this ionization technique to a wider variety of compounds. In this work, a novel identification method was developed using an LC-LEI-QTOF-MS/MS system, adding the high-resolution mass spectrometry (HRMS) capabilities to EI identification power. This approach facilitated the characterization of volatile, low volatile, and thermolabile fractions of 13 CF formulations with high levels of confidence due to the comparison with the NIST library. Seven compounds were easily identified with high confidence levels, 1 or 2a. In addition to the NIST comparison, low energy analysis confirmed compound identities, determining molecular ions. Differently from other published studies performed using EI, where the molecular ion was established using the CI ion source [32], in this work the molecular ions were determined without the need for other instruments or ion sources by varying the electron energy. The identities were also confirmed by the accurate masses and HR molecular ion fragmentations. The library did not recognize four compounds, but their structures were proposed with high scores by the MSC software, and the satisfactory accurate masses allow their identification with a confidence level of 2b. For three compounds, the molecular formulas were proposed with good accuracy, and for only one compound it was not possible to establish the possible formula, perhaps due to its low concentration. In the UA_3 formulation, various contaminants were identified, including unexpected ones such as the banned pesticide Carbosulfan, its toxic metabolite Dibutylamine, and its impurity C_24_H_28_N_2_O_6_S_2_. Other notable findings across formulations included DEET, a common mosquito repellent; C_14_H_18_N_2_O_4_; and 3-keto-carbofuran, a primary metabolite of carbofuran. A custom high-resolution spectral library simplified the detection of these compounds in real samples, even in cases of low concentrations or matrix coelution. 3-keto-carbofuran, present in one formulation (F0), was detected alongside CF in the fox liver sample (544/2). However, as a metabolite of carbofuran, 3-keto-carbofuran cannot serve as a marker for a specific carbofuran formulation.

Regarding the quantitative analysis, the method demonstrated excellent performance. As expected, negligible *ME* were confirmed also without the use of an internal standard, confirming that the LC-LEI-QTOF-MS/MS system is a good alternative for qualitative and quantitative analysis of volatile and low-volatile fractions of complex samples.

Although the recovery experiments were not performed, and the concentrations found in CF-positive samples could be underestimated, their real values were well above the LD50 values in rats (5 mg/kg) and birds (0.7–8.0 mg/kg), and comparable with values reported in human suicide cases, confirming the suspected intentional poisoning.

## Figures and Tables

**Table 1:** List of CF formulations analyzed with the number of identified compounds.

Formulation Name	Status	Number of Putatively Identified Compounds
UA_3	liquid	12
BV_PF_B	solid	2
CG_23_B	solid	2
F_0	solid	2
F_2	solid	1
F_8	solid	1
F_9	solid	1
CG_23_A	solid	1
UA_1	liquid	1
F_8	solid	1
F_9	solid	1
CG_23_A	solid	1
UA_1	liquid	1
UA_2	liquid	1
BV_PF_A	solid	1
Cd	solid	1
SL	solid	1

**Table 2:** Peaks list of UA_3 formulation.

Peak	RT	Molecular Ion (*m*/*z*)	Suspected Compound	dM (PPM)	NIST Match (%)	Confidence Level
1	5.35	129.1507	Dibutylamine	−3.8	57.21	2b
2	7.69	221.1043	Carbofuran	−1.6	96.37	1
3	8.83	128.0623	Naphtalene	−1.9	99.38	1
4	9.09	118.0772	Indane	−4.2	92.91	2a
5	9.19	142.0772	2-Methylnaphthalene	−2.8	/	2b
6	9.25	120.0935	Mesitylene	1.2	96.48	2a
7	9.39	132.0935	Indan-1-Methyl	1.1	94.66	2a
8	9.51	134.1093	Cymene	2.2	90.11	2a
9	9.72	170.1082	2,3,6-Trimethylnaphtalene	4.7	/	2b
10	9.87	504.1400	C_24_H_28_N_2_O_6_S_2_	3.3	/	4
11	10.46	381.2206 (M+H)^+^	Carbosulfan	−1.15	76.74	2b
12	11.18	/	/	/	/	/

**Table 3:** Peaks list of BV_PF_B formulation.

Peak	RT	Molecular	Suspected Compound	dM (PPM)	NIST	Confidence
		Ion (*m*/*z*)			Match (%)	Level
1	7.49	221.1043	Carbofuran	−1.6	95.68	1
2	7.69	191.1291	DEET	−7.14	91.84	1

**Table 4:** Peaks list of CG_23_B formulation.

Peak	RT	Molecular	Suspected	dM	NIST	Confidence
		Ion (*m*/*z*)	Compound	(PPM)	Match (%)	Level
1	7.52	221.1043	Carbofuran	−1.6	95.68	1
2	7.76	278.1258	C_14_H_18_N_2_O_4_	−1.1	/	4

**Table 5:** Peak list of F0.

Peak	RT	Molecular Ion (*m*/*z*)	Suspected Compound	dM (PPM)	NIST Match (%)	Confidence Level
1	6.80	235.0952	3-keto-carbofuran	−0.9	62.85	4
2	7.69	221.1043	Carbofuran	−1.6	95.68	1

## Data Availability

The original contributions presented in this study are included in the article and Supplementary Materials. Further inquiries can be directed to the corresponding author.

## Supplementary Materials

The following supporting information can be downloaded at: https://www.mdpi.com/article/10.3390/molecules31020259/s1, Figure S1. (A–C): Comparison with NIST library; Figure S2. (A–C): Comparison with NIST library; Figure S3. (A–C): Comparison with NIST library; Figure S4. (A–C): Comparison with NIST library; Figure S5. (A–C): Comparison with NIST library; Figure S6. (A–C): Comparison with NIST library; Figure S7. (A,B): Structure identification using molecular structure correlator software; Figure S8. (A,B): Structure identification using molecular structure correlator software; Figure S9. (A,B): Molecular formula determination using the molecular ion accurate mass; Figure S10. (A,B): Comparison with NIST library; Figure S11. (A–C): Comparison with NIST library.

## Abbreviations

The following abbreviations are used in this manuscript:

CF	Carbofuran
GC	Gas Chromatography
LC	Liquid Chromatography
MS	Mass Spectrometry
EI	Electron Ionization
ESI	Electrospray Ionization
HRMS	High Resolution Mass Spectrometry
LEI	Liquid Electron Ionization
NTA	Non-Targeted Analysis
*ME*	Matrix Effects
Q-TOF	Quadrupole Time-Of-Flight
PLE	Pressurized Liquid Extraction
PFS	Passive Flow Splitter
VMC	Vaporization Microchannel
MSC	Molecular Structure Correlator Software
