# Supplementary material for: Non-Targeted Analysis of Carbofuran and Related Compounds in Commercial Formulations and Animal Tissue
Source: Molecules. 2026 Jan 12;31(2):259. doi: 10.3390/molecules31020259 (PMC12844122; doi:10.3390/molecules31020259)
Supplement: Supplementary file 1 [file molecules-31-00259-s001.zip › molecules-4027317-supplementary.pdf]

Non-Targeted Analysis of Carbofuran and Related Compounds in Commercial Formulations and Animal Tissue

Genny Grasselli[a], Adriana Arigò[a], Giorgio Famiglini[a], Zdena Skrob[b], Arthur Sniegon[b], Tomas Cajthaml[b], and Achille Cappiello[a c] *

[a] *Department of Pure and Applied Sciences, University of Urbino Carlo Bo, 61029, Urbino, Italy*

[b] *Institute of Environmental Studies, Faculty of Sciences, Charles University, 12801, Prague, Czech Republic*

[c] *Vancouver Island University, Nanaimo, BC, V9R 5S5, Canada.*

* Email: achille.cappiello@uniurb.it

Summary:

Contents

Figure S1 (A, B, C): Comparison with NIST library.

Figure S2 (A, B, C): Comparison with NIST library.

Figure S3 (A, B, C): Comparison with NIST library.

Figure S4 (A, B, C): Comparison with NIST library.

Figure S5 (A, B, C): Comparison with NIST library.

Figure S6 (A, B, C): Comparison with NIST library.

Figure S7 (A, B): Structure identification using molecular structure correlator software.

Figure S8 (A, B): Structure identification using molecular structure correlator software.

Figure S9 (A, B): Molecular formula determination using the molecular ion accurate mass.

Figure S10 (A, B): Comparison with NIST library.

Figure S11 (A, B, C): Comparison with NIST library.

Compound Name	Match Factor	CAS#
Carbofuran	96.37	1563-66-2
7-Benzofuranol, 2...	95.59	1563-38-8
7-Benzofuranol, 2...	95.32	1563-38-8
Carbofuran	94.96	1563-66-2
Carbofuran	94.52	1563-66-2
Carbofuran	93.90	1563-66-2
Carbofuran	93.30	1563-66-2
7-Benzofuranol, 2...	92.78	1563-38-8
2,4,6-Cycloheptat...	87.11	672-76-4
Silane, trimethyl(p...	85.26	770-09-2

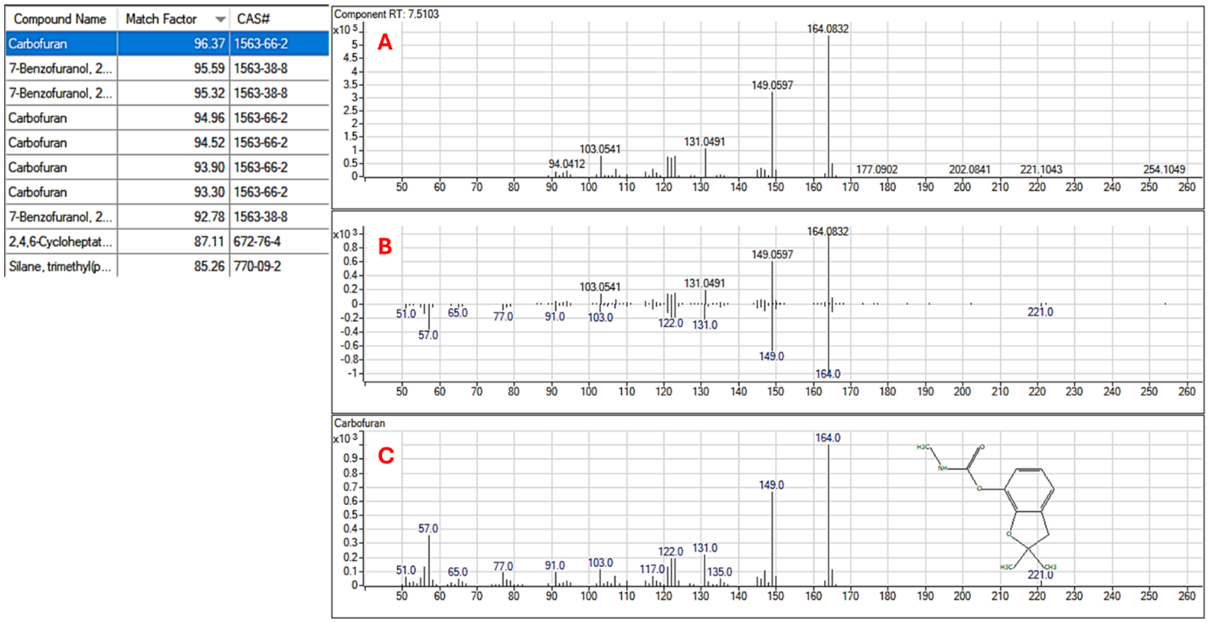

Figure S1 (A,B,C). A: Experimental spectrum of Carbofuran; **B:** Comparison between experimental spectrum and NIST library spectrum; **C:** NIST library Carbofuran spectrum.

Compound Name	Match Factor	CAS#
Naphthalene	99.38	91-20-3
Azulene	99.25	275-51-4
Naphthalene	98.72	91-20-3
Naphthalene	98.36	91-20-3
Azulene	97.51	275-51-4
Azulene	97.25	275-51-4
Azulene	97.10	275-51-4
Naphthalene	96.89	91-20-3
1H-Indene, 1-met...	96.75	2471-84-3
[4.2.2]Propella-2,...	93.49	88090-34-0

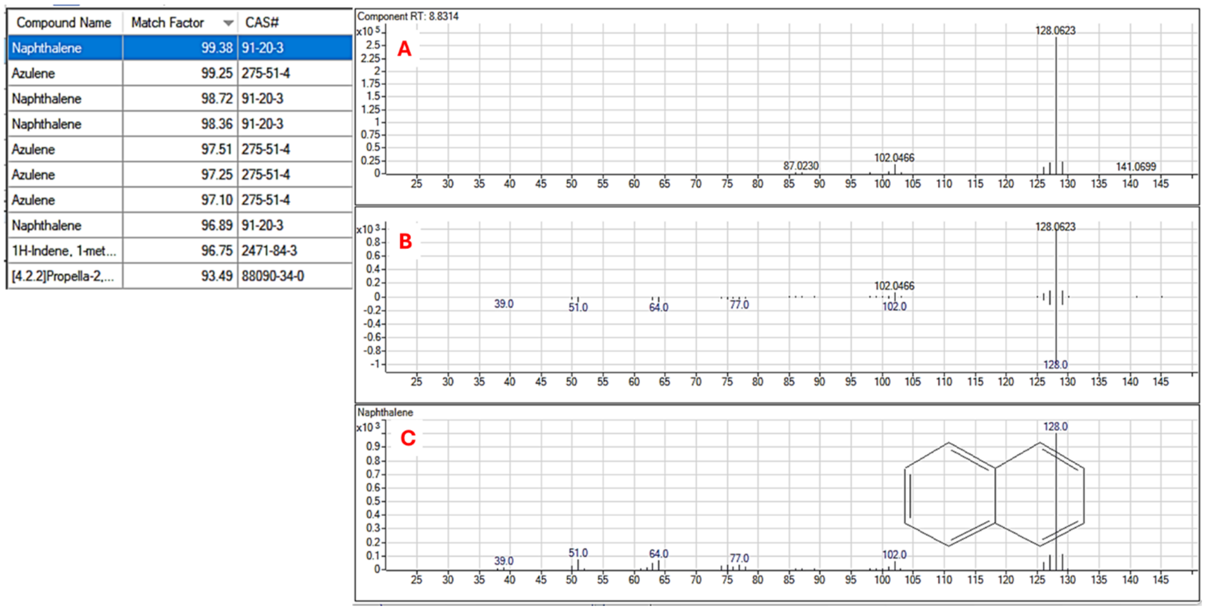

Figure S2 (A,B,C). A: Experimental spectrum of Naphthalene; **B:** Comparison between experimental spectrum and NIST library spectrum; **C:** NIST library Naphthalene spectrum.

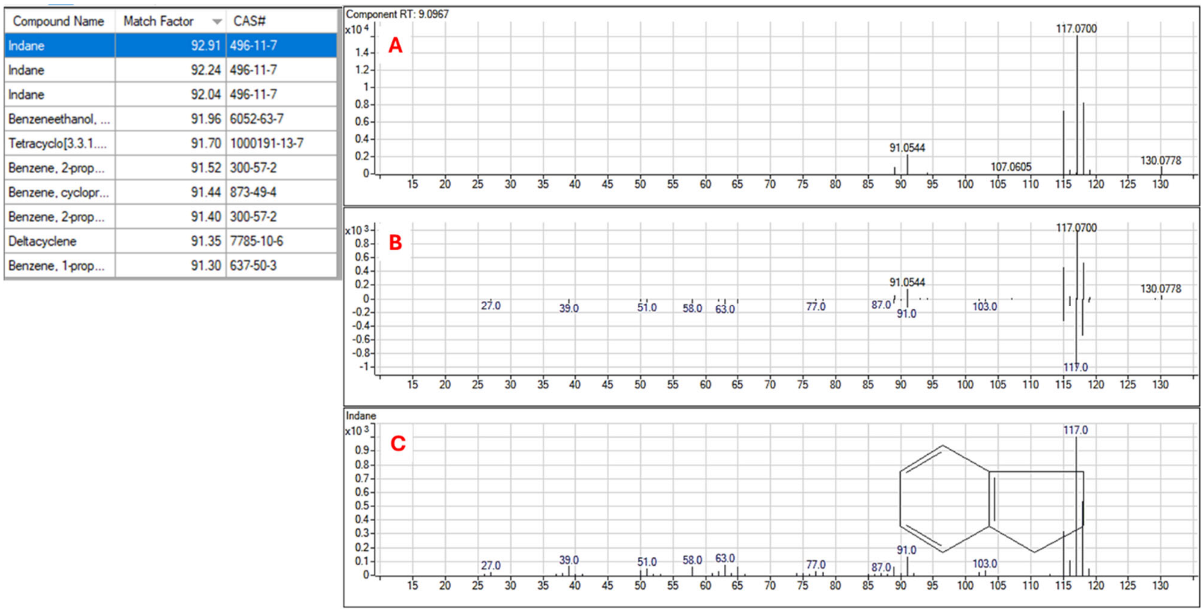

Figure S3 (A,B,C). A: Experimental spectrum of Indane; **B:** Comparison between experimental spectrum and NIST library spectrum; **C:** NIST library Indane spectrum.

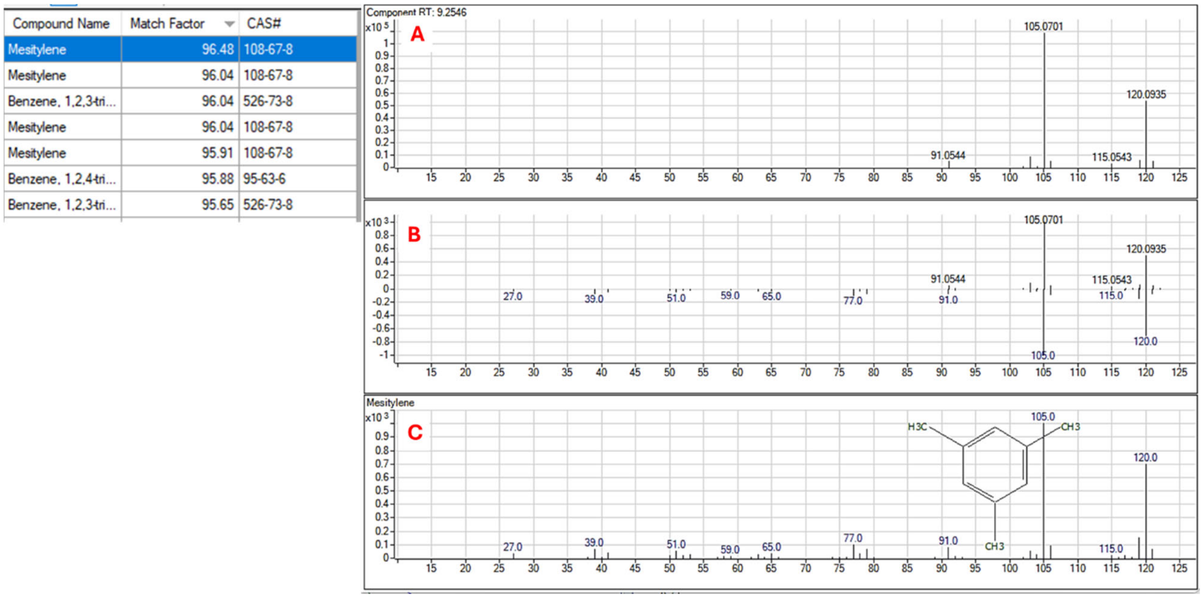

Figure S4 (A,B,C). A: Experimental spectrum of Mesitylene; **B:** Comparison between experimental spectrum and NIST library spectrum; **C:** NIST library Mesitylene spectrum.

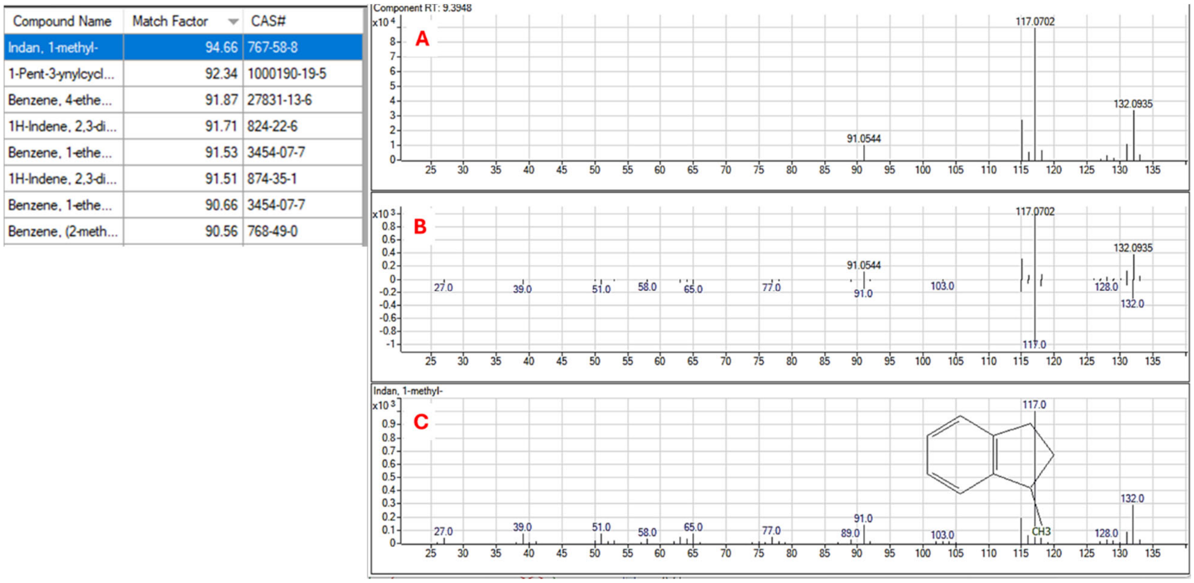

Compound Name	Match Factor	CAS#
Indan, 1-methyl-	94.66	767-58-8
1-Pent-3-ynylcycl...	92.34	1000190-19-5
Benzene, 4-ethe...	91.87	27831-13-6
1H-Indene, 2,3-di...	91.71	824-22-6
Benzene, 1-ethe...	91.53	3454-07-7
1H-Indene, 2,3-di...	91.51	874-35-1
Benzene, 1-ethe...	90.66	3454-07-7
Benzene, (2-meth...	90.56	768-49-0

Figure S5 (A,B,C). A: Experimental spectrum of Indane-1-Methyl; **B:** Comparison between experimental spectrum and NIST library spectrum; **C:** NIST library Indane-1-Methyl spectrum.

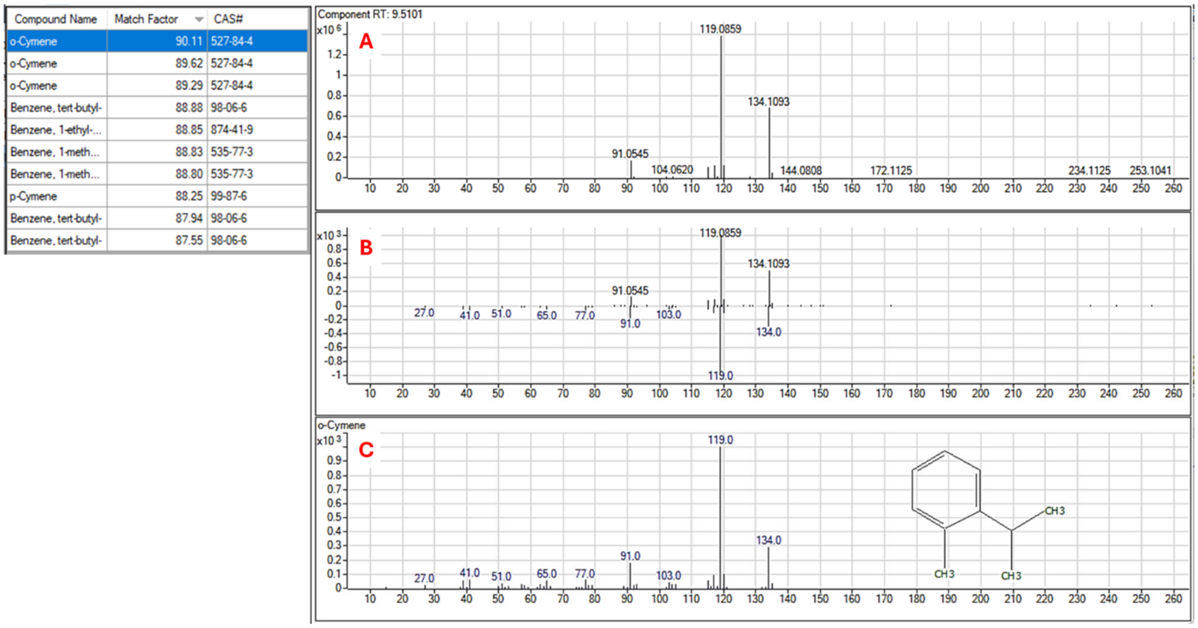

Compound Name	Match Factor	CAS#
o-Cymene	90.11	527-84-4
o-Cymene	89.62	527-84-4
o-Cymene	89.29	527-84-4
Benzene, tert-butyl-	88.88	98-06-6
Benzene, 1-ethyl-...	88.85	874-41-9
Benzene, 1-meth...	88.83	535-77-3
Benzene, 1-meth...	88.80	535-77-3
p-Cymene	88.25	99-87-6
Benzene, tert-butyl-	87.94	98-06-6
Benzene, tert-butyl-	87.55	98-06-6

Figure S6 (A,B,C). A: Experimental spectrum of Cymene; **B:** Comparison between experimental spectrum and NIST library spectrum; **C:** NIST library Cymene spectrum.

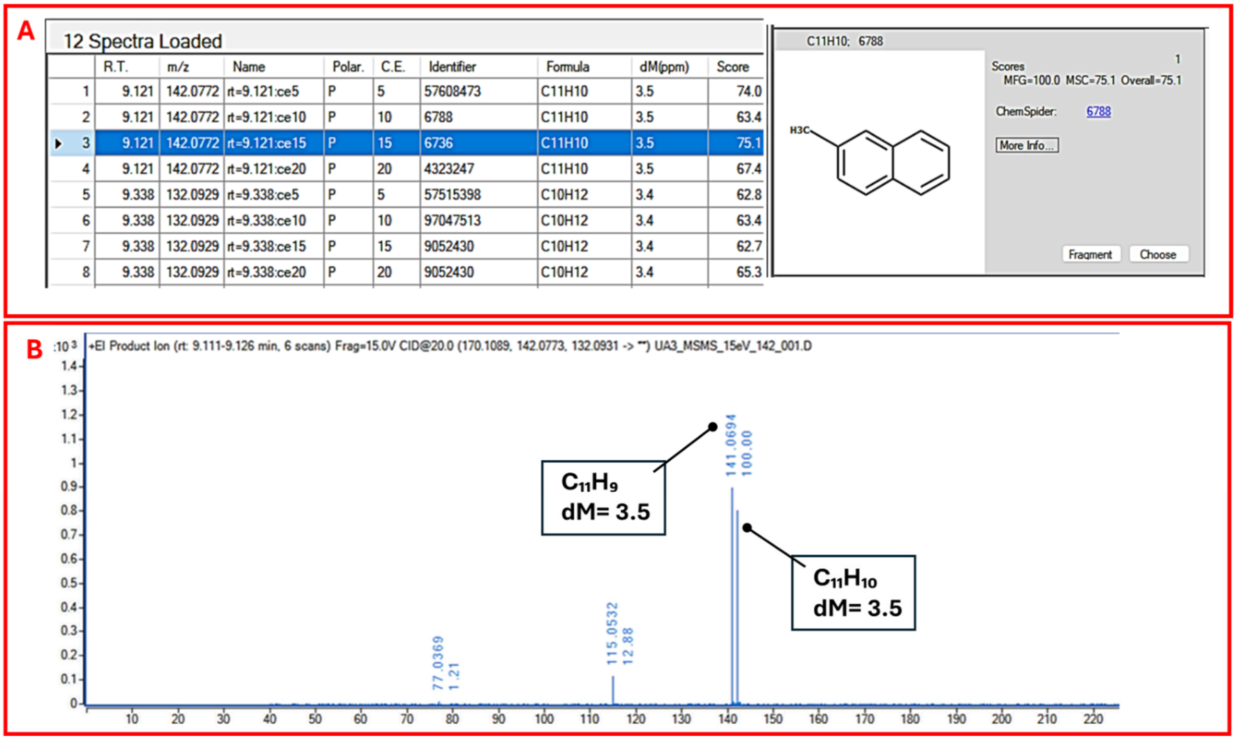

Figure S7 (A,B). A: Identification of 2-Methylnaphtalene (peak 5) using molecular structure correlator software; **B:** MS/MS fragmentation of 2-Methylnaphtalene molecular ion (m/z 142.0772).

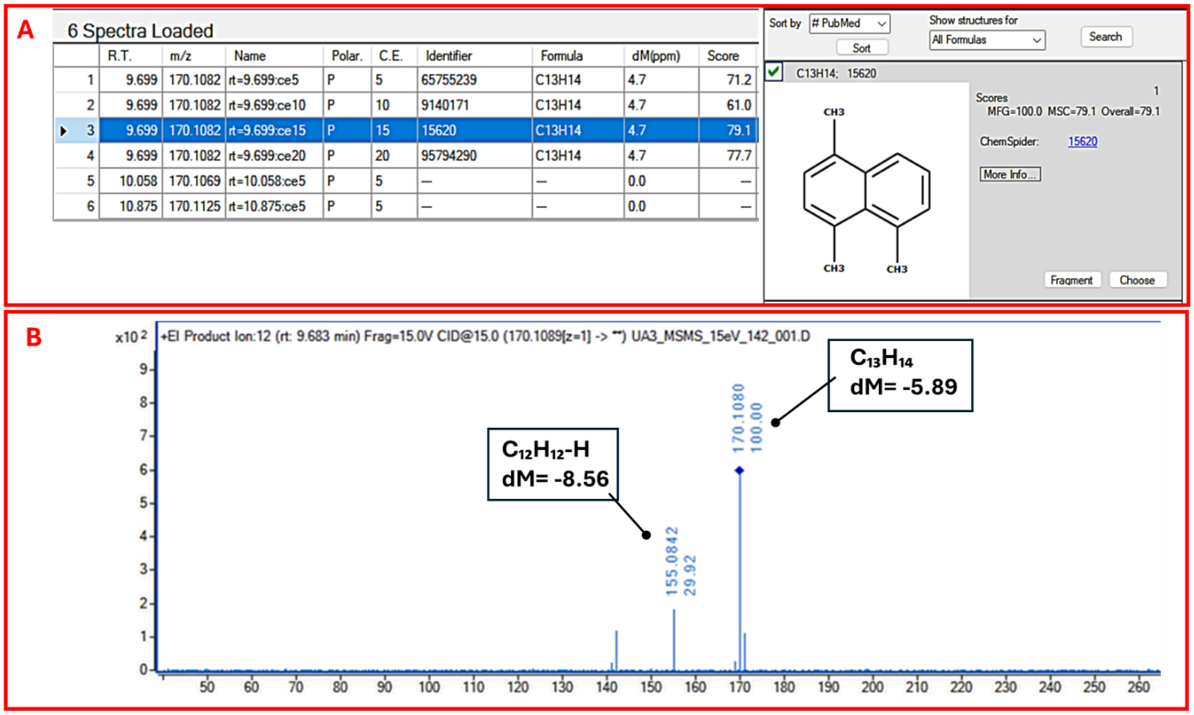

Figure S8 (A,B). A: Identification of 2,3,6-Trimethylnaphtalene (peak 9) using molecular structure correlator software; **B:** MS/MS fragmentation of 2,3,6-Trimethylnaphtalene molecular ion (m/z 170.1080).

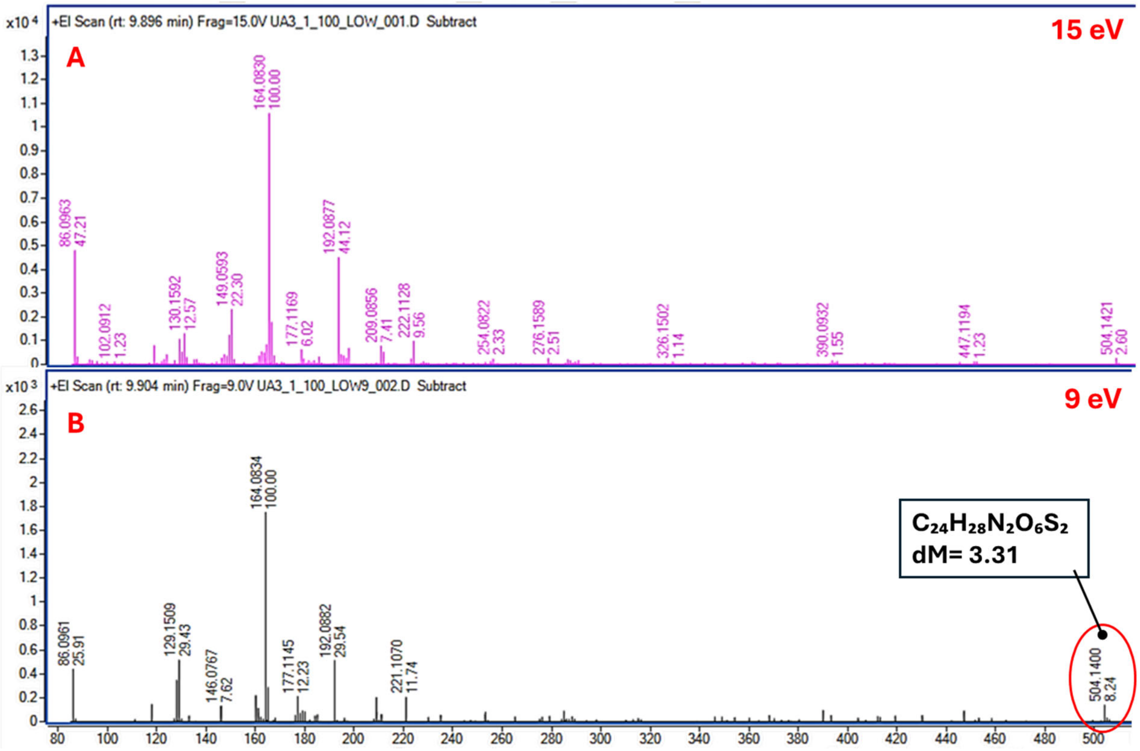

Figure S9 (A,B). 15 eV **(A)** and 9 eV **(B)** spectra of $C_{24}H_{28}N_2O_6S_2$ (peak 10) and, in red, the suspected molecular ion.

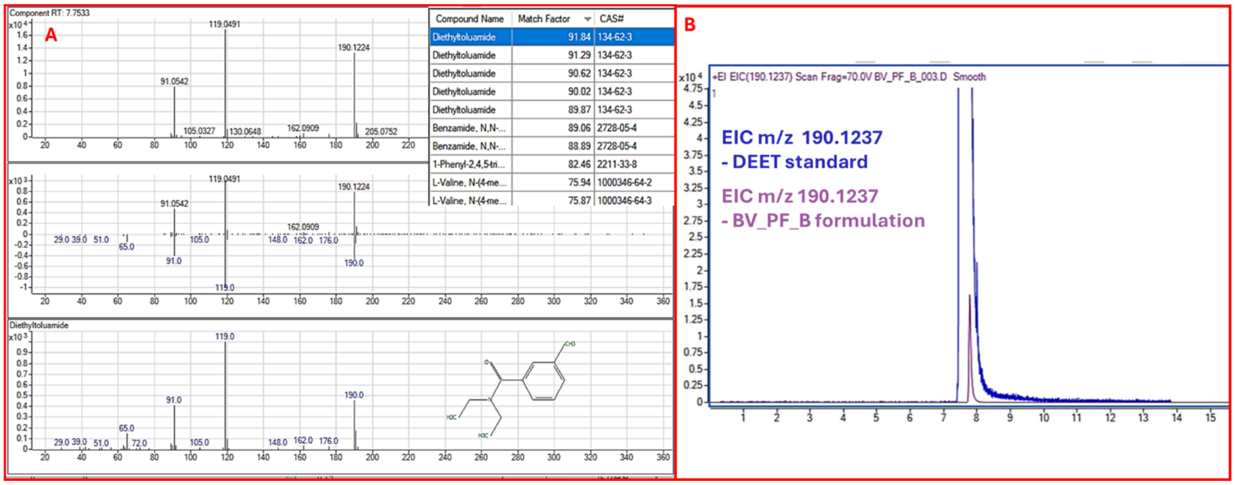

Figure S10 (A,B). A: Comparison between NIST library and experimental DEET (peak 8) spectrum; **B:** RT comparison between DEET peak of standard (blue) and in BV_PF_B formulation.

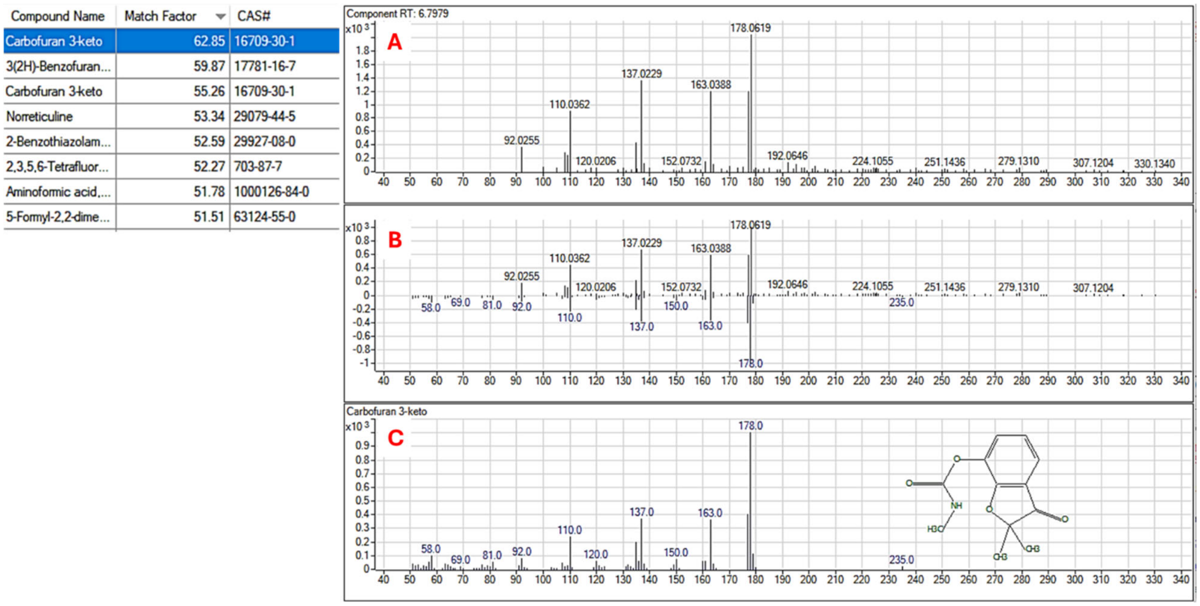

Figure S11 (A,B,C). A: Experimental spectrum of 3-keto-carbofuran (peak 1, formulation F0) spectrum**; B:** Comparison between experimental spectrum and NIST library spectrum; **C:** NIST library Carbofuran 3-keto spectrum.
